# The crystal structure of the deca­aluminum alkoxide cluster Al_10_O_4_(OH)_8_ *L*_14_ (*L* = 1,1,1,3,3,3-hexa­fluoro­propan-2-olate)

**DOI:** 10.1107/S2056989020016618

**Published:** 2021-01-05

**Authors:** Ray J. Butcher, Andrew P. Purdy

**Affiliations:** a Department of Chemistry, Howard University, 525 College Street NW, Washington DC 20059, USA; b Chemistry Division, Code 6123, Naval Research Laboratory, 4555 Overlook Av, SW, Washington DC 20375-5342, USA

**Keywords:** crystal structure, 1,1,1,3,3,3-hexa­fluoro­propan-2-olate ligand, aluminium cluster

## Abstract

In the title centrosymmetric cluster compound, hexa­kis­(μ_2_-1,1,1,3,3,3-hexa­fluoro­propan-2-olato)octa­kis­(1,1,1,3,3,3-hexa­fluoro­propan-2-olato)octa-μ_2_-hydroxido-di-μ_4_-oxido-di-μ_3_-oxido-deca­aluminium, [Al_10_(C_3_HF_6_O)_14_(OH)_8_O_4_] (C_42_H_22_Al_10_F_84_O_26_), there is a central μ_4_-OAl_4_ moiety, which has six edges of which three contain μ(*O*)-1,1,1,3,3,3-hexa­fluoro­propan-2-olate (*L*) ligands and two contain μ-OH groups each bridging two Al atoms along an edge. The sixth edge is occupied by a group containing a fifth aluminium atom [bis-μ(OH)-, μ_3_(O)—Al*L*]. This last μ_3_(O) group generates a centrosymmetric Al_2_O_2_ dimer, thus the μ_3_(O) atom is linked to two Al atoms in the asymmetric unit as well as a third Al atom through a center of inversion. Three of the hexa­fluoro­propyl groups of the C_3_HF_6_O^−^ ligands are disordered and each was refined with two conformations with occupancies of 0.770 (3)/0.230 (3), 0.772 (3)/0.228 (3) and 0.775 (3)/0.225 (3). The five unique Al centers have coordination numbers varying from four to six with bond angles that show considerable distortions from regular geometry: for the four-coordinate atom, τ_4_′ = 0.886, while three Al atoms are five-coordinate (τ_5_ values = 0.098, 1.028, and 0.338) and one is distorted six-coordinate with O—Al—O bond angles ranging from 74.22 (9) to 171.59 (12)°. The geometry about the central O atom in the OAl_4_ block is significantly distorted tetra­hedral (τ_4_′ = 0.630) with Al—O—Al angles ranging from 95.50 (9) to 147.74 (13)°. The extended structure features numerous O—H⋯O, O—H⋯F, C—H⋯O and C—H⋯F hydrogen bonds and short F⋯F contacts.

## Abstract 2

The title centrosymmetric deca­aluminum cluster, Al_10_O_4_(OH)_8_(C_3_HF_6_O)_14_, contains two OAl_4_ units and a central Al_2_(O)_2_ bridge.

## Chemical context   

The inter­est in metal alkoxides (Turova *et al.*, 2002 ▸) is due to their potential use as precursors of oxide materials in sol–gel technology (Brinker & Scherer, 1990 ▸) with applications in many fields including biomaterials (Avnir *et al.*, 2006 ▸), and in the synthesis of single-phase materials, which provide unique possibilities to tailor the mechanical, electrical, and optical properties (Schottner, 2001 ▸). Within this class of compounds, the alkoxides of aluminum are of great inter­est and the first aluminum compounds with monodentate alkoxide ligands have been known since 1881. However, in spite of this inter­est, there are few examples of simple monodentate aluminum alkoxides that have been structurally characterized by single crystal X-ray analysis. In order of complexity, the dinuclear structure, Al_2_(O*t*Bu)_6_ [*t*Bu = *tert*-but­yl], was published in 1991 (Cayton *et al.*, 1991 ▸) followed by trinuclear Al_3_(OCHex)_9_ [CHex = cyclo­hex­yl] in 2000 (Pauls & Neumüller, 2000 ▸). The crystal structure of the tetra­nuclear compound Al_4_(O*i*Pr)_12_ [*i*Pr = isoprop­yl] was first reported in 1979 (Turova *et al.*, 1979 ▸) and re-determined in 1991 (Folting *et al.*, 1991 ▸). An additional structure with four Al atoms and containing a μ_4_-O atom bridging all four Al atoms, [Al_4_(OCH_2_CF_3_)_11_]^−^ (one H atom could not be located) has been reported (Sangokoya *et al.*, 1993 ▸). A penta­nuclear, Al_5_O(O*i-*Bu)_13_, and octa­nuclear structure, Al_8_O_2_(OH)_2_(O*i*Bu)_18_ (*i*Bu = *iso*-but­yl), was determined in 2002 (Abrahams *et al.*, 2002 ▸). In 2018, the structure of a nona­nuclear structure, Al_9_O_3_(OEt)_21_, was reported (Nachtigall *et al.* 2018 ▸). In 1987, the deca­nuclear compound, Al_10_O_4_(OEt)_22_, was reported (Yanovsky *et al.*, 1987 ▸). The polynuclear aluminum oxoalkoxide structure containing the largest number of Al atoms solely from simple alcohols reported to date was Al_11_O_6_(O*n*Pr)_10_(O*i*Pr)_10_(O*i*/*n*Pr)(HO*i*/*n*Pr)_2_ (*n*Pr = *n*-prop­yl) in 2004 (Starikova *et al.*, 2004 ▸). In a continuation of these studies, the structure of the complex derived from perfluorinated 2-propanol and aluminum ions, Al_10_O_4_(OH)_8_ *L*_14_ [*L* = 1,1,1,3,3,3-hexa­fluoro­propan-2-olate], **1**, is now reported.
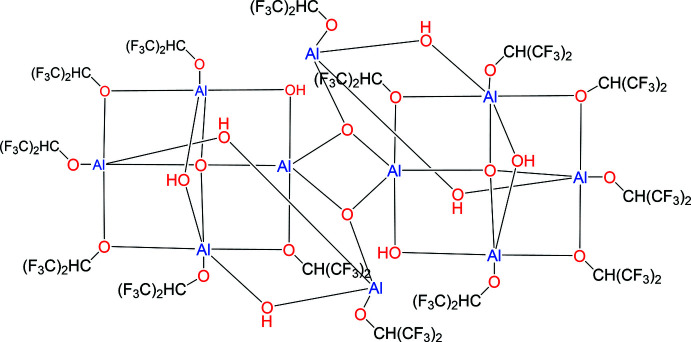


## Structural commentary   

The structure of the title compound (C_42_H_22_Al_10_F_84_O_26_) is best described in terms of its building blocks. First there is a μ_4_-OAl_4_ moiety (O1, Al1–Al4), which has six edges of which three contain μ(O)-1,1,1,3,3,3-hexa­fluoro­propan-2-olate (*L*) ligands and two contain μ-OH groups, each bridging two Al atoms along an edge (Al1–Al2, Al2–Al4, and Al3–Al4 for *L* and Al1–Al3 and Al2–Al3 for the μ-OH groups). The sixth edge (Al1–Al4) is occupied by a group containing a fifth Al atom [bis-μ(OH)-, μ_3_(O)-Al*L*] where one μ(OH) bridges Al4–Al5 and the μ_3_(O) group bridges A11–Al5, while the second μ(OH) bridges Al2–Al5. This last μ_3_(O) group allows this overall moiety to form a centrosymmetric Al_2_O_2_ deca-aluminum dimer, thus each μ_3_(O) group is linked to Al1 and Al5 in the asymmetric unit as well as a second Al1 atom through a center of inversion (symmetry operation −*x*, 1 − *y*, 1 − *z*).

Apart from the simpler homoleptic aluminum alkoxides containing two, three, and four aluminum atoms, in the larger aggregates the important building block appears to be a central O atom surrounded by four Al atoms in a distorted tetra­hedral arrangement, *i.e*. OAl_4_ [five Al atoms in the case of Al_5_O(O*i-*Bu)_13_ (Abrahams *et al.*, 2002 ▸) but this is an exception and also not an aggregate]. In each case in this OAl_4_ building block, five of the six edges are occupied by a μ(O)-alkoxide bridge while the sixth edge is vacant to allow for dimerization. In larger aggregates, in the case of Al_8_O_2_(OH)_2_(O*i*Bu)_18_ (Abrahams *et al.*, 2002 ▸), these building blocks are linked by two μ-OH units. For Al_9_O_3_(OEt)_21_ (Nachtigall *et al.* 2018 ▸), these building blocks are linked by two moieties. The first is a μ_3_(O) group linking the two halves as well as the ninth Al atom. The second link is provided by a central Al(OEt)_4_ group, which links the two building blocks through two μ(OEt) on each side of the ninth Al atom. In the case of Al_10_O_4_(OEt)_22_ (Yanovsky *et al.*, 1987 ▸), these units are again linked by two moieties somewhat analogous to the situation for Al_9_O_3_(OEt)_21_. Both contain a μ_3_(O) group linking the two halves as well as an additional Al(OEt)_4_ group, which links the two building blocks through two μ(OEt) on each side of the group. However, in this instance this both linking moieties are located about a center of inversion The situation for Al_11_O_6_(O*n*Pr)_10_(O*i*Pr)_10_(O*i*/*n*Pr)(HO*i*/*n*Pr)_2_ (Starikova *et al.*, 2004 ▸) is slightly more complex: in this case the two building blocks are linked by group containing three Al atoms of which the central Al is located on a twofold crystallographic axis. This central Al is linked to both the O_4_Al building blocks and the other Al in the linking moiety by both two μ_2_(O) and μ_3_(O) linkages and also contains a terminal OEt ligand.

From this survey of aluminum alkoxide aggregates containing more than five Al centers, it can be seen that the present structure is unique in both its building block and the method of aggregation. In this instance, the edges of the OAl_4_ block are made up by three μ(O)-1,1,1,3,3,3-hexa­fluoro­propan-2-olate (*L*) and two μ-OH bridges with the sixth edge vacant to allow for dimerization. Aggregation is achieved by a μ_3_(O) group as in the other cases as well as a Al(OH)_2_(O)(*L*) moiety containing both μ(OH) and μ(O) groups where the latter are used to achieve dimerization.

**Figure 1:**
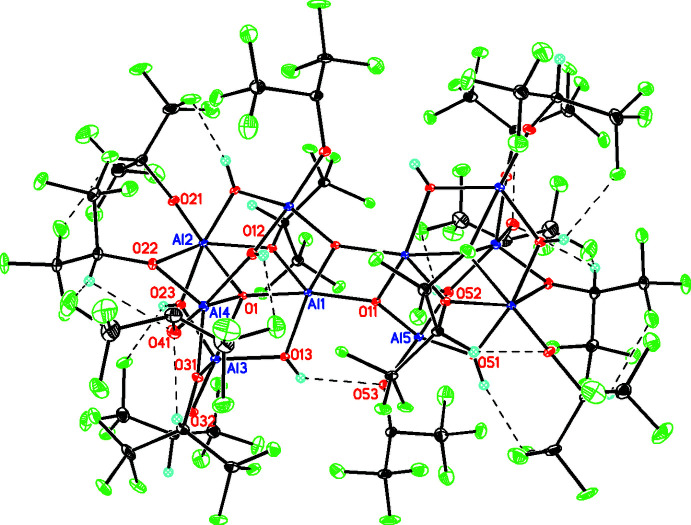
The mol­ecular structure of the deca­aluminium cluster in **1** showing labeling for Al and O only for clarity (major component only; unlabeled atoms are generated by −*x*, 1 − *y*, 1 − *z*). Atomic displacement parameters are shown at the 30% probability level. Intra­molecular O—H⋯O, O—H⋯F and C—H⋯F inter­actions are shown by dashed lines.

**Figure 2:**
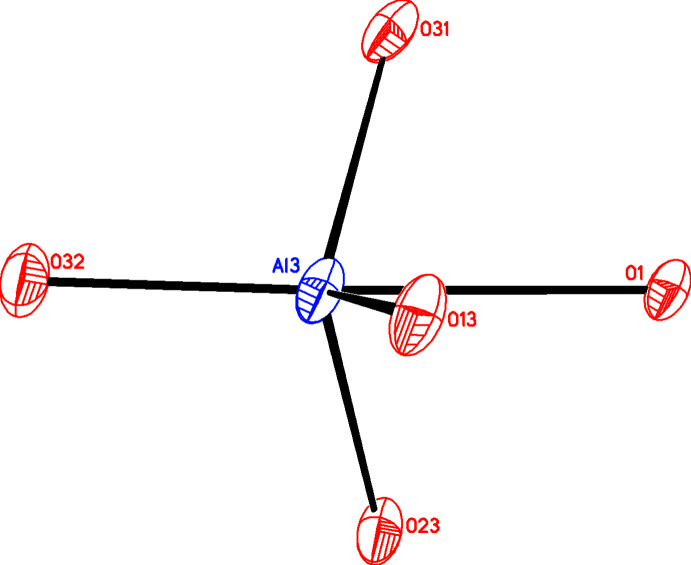
Diagram showing the five-coordinate environment about Al3 in which the metal ion is displaced out of the trigonal plane leading to a τ_5_ value of 1.028 (> 1).

Typically the Al centers in these aluminum alkoxide aggregates have varying coordination numbers from four to six with angles that vary widely from regular geometry and this is true in **1** (Table 1 ▸ and Fig. 1 ▸) where Al5 is four-coordinate [τ_4_′ = 0.886 (Okuniewski *et al.*, 2015 ▸) indicating slightly distorted tetra­hedral], while Al1, Al3, and Al4 are all five-coordinate [τ_5_ values are 0.098, 1.028, and 0.338, respectively (Addison *et al.*, 1984 ▸)] while Al2 is distorted six-coordinate with O—Al—O bond angles ranging from 74.22 (9) to 171.59 (12)°. A τ_5_ value of 1.028 is outside the normal range from 0 to 1 (Addison *et al.*, 1984 ▸) so some comment should be made. A recent paper (Blackman *et al.*, 2020 ▸) gave examples of this situation in which the geometries were all distorted trigonal pyramidal with the metal out of the trigonal plane, as is the case for Al3 (Fig. 2 ▸). The geometry about the central O atom in the OAl_4_ block is significantly distorted tetra­hedral [τ_4_′ = 0.630 (Okuniewski *et al.*, 2015 ▸)] with Al—O—Al angles ranging from 95.50 (9) to 147.74 (13)°.

## Supra­molecular features   

**Figure 3:**
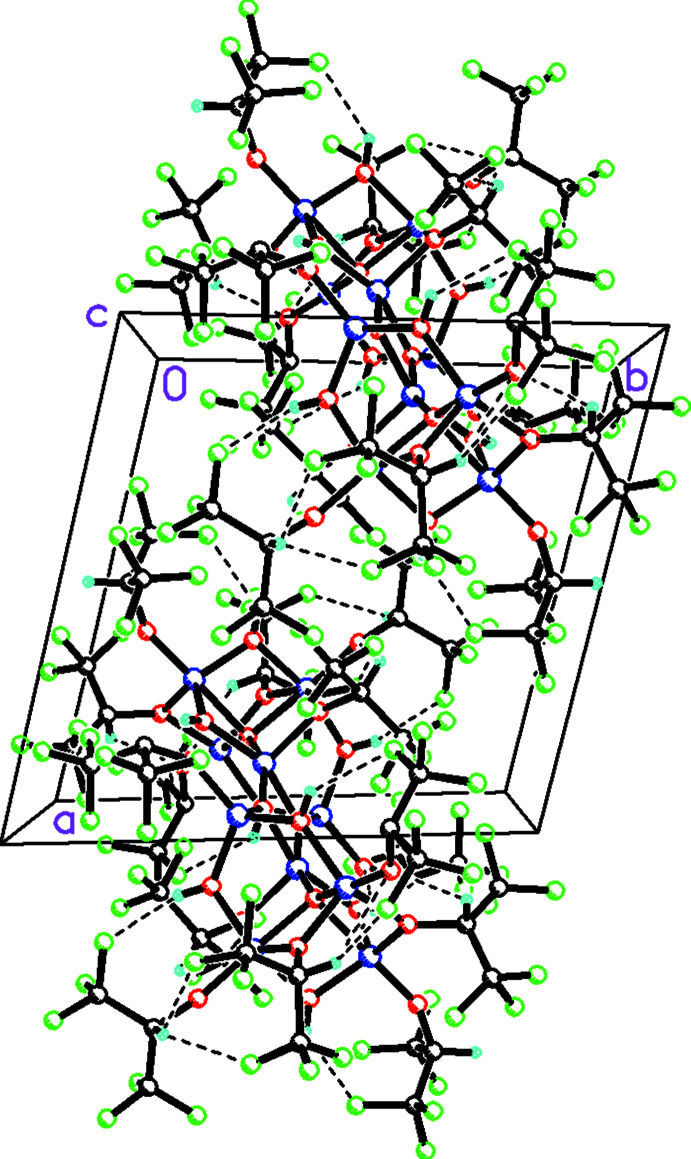
Packing diagram of the deca­aluminium cluster in **1** viewed along the *c*-axis direction. Inter-cluster F⋯F inter­actions and both intra-cluster and inter-cluster O—H⋯O, O—H⋯F and C—H⋯F inter­actions are shown with dashed lines.

In the extended structure of **1**, the deca-aluminum clusters make numerous inter­molecular F⋯F contacts, which are less than the sum of their van der Waals (Alvarez, 2013 ▸) radii, ranging in length from 2.641 (4) [F143⋯F211(1 − *x*, 2 − *y*, 1 − *z*) to 2.921 (4) Å [F31⋯F202(*x*, −1 + *y*, *z*) (see Fig. 3 ▸). In addition there are strong O—H⋯O and O—H⋯F and weak C—H⋯O and C—H⋯F hydrogen bonds, which help to consolidate the aluminum aggregates (Table 2 ▸).

## Database survey   

A search of the Cambridge Structural Database [CSD version 5.41 (November 2019); Groom *et al.*, 2016 ▸] for fragments based on the structure of **1** gave five hits [ERUBEY (Starikova *et al.*, 2004 ▸); QESHOO (Nachtigall *et al.* 2018 ▸); UDOTAI and UDOTEM (Abrahams *et al.*, 2002 ▸) and ZZZGIE11 (Yanovsky *et al.*, 1987 ▸)]. A survey of the literature also revealed other structures not found from this search (Cayton *et al.*, 1991 ▸; Pauls & Neumüller, 2000 ▸; Folting *et al.*, 1991 ▸; Sangokoya *et al.*, 1993 ▸).

## Synthesis and crystallization   

A solution of Al(BH_4_)_3_ (Olson and Sanderson, 1958 ▸) in toluene was prepared by a reaction of AlCl_3_ with 3 eq. of LiBH_4_ in toluene, followed by distillation. In a bulb, 21.18 mmol of hexa­fluoro­iso­propanol were condensed into 1.76 mmol of Al(BH_4_)_3_ solution in several portions, and allowed to react to completion. Two phases formed, and then the second phase redissolved. The yellow liquid product was stored in a vial in a dry box, and on a day where the room temperature was very cold (<15 °C), colorless crystals formed. The crystals quickly melt at normal room temperature, and had to be placed into the cold stream immediately upon isolation.

## Refinement   

Crystal data, data collection and structure refinement details are summarized in Table 3 ▸. Several of the hexa­fluoro­propyl groups are disordered and each was refined with two equivalent conformations with occupancies of 0.770 (3)/0.230 (3), 0.772 (3)/0.228 (3) and 0.775 (3)/0.225 (3). The H atoms attached to C were refined in idealized positions using a riding model with C—H = 1.00 Å and *U*_iso_(H) = 1.2*U*_eq_(C), while those attached to O were refined isotropically.

## Supplementary Material

Crystal structure: contains datablock(s) I, global. DOI: 10.1107/S2056989020016618/hb7956sup1.cif

Structure factors: contains datablock(s) I. DOI: 10.1107/S2056989020016618/hb7956Isup2.hkl

CCDC reference: 2052015

Additional supporting information:  crystallographic information; 3D view; checkCIF report

## Figures and Tables

**Table 1:** Selected bond lengths (Å)

Al1—O11	1.781 (2)	Al3—O13	1.803 (2)
Al1—O13	1.833 (2)	Al3—O31	1.856 (3)
Al1—O12	1.839 (2)	Al3—O1	2.034 (2)
Al1—O11^i^	1.839 (2)	Al4—O41	1.734 (3)
Al1—O1	1.852 (2)	Al4—O22	1.830 (2)
Al2—O21	1.729 (2)	Al4—O1	1.831 (2)
Al2—O23	1.861 (2)	Al4—O52^i^	1.872 (2)
Al2—O51^i^	1.893 (2)	Al4—O31	1.932 (2)
Al2—O1	1.900 (2)	Al5—O53	1.714 (2)
Al2—O12	2.023 (2)	Al5—O11	1.734 (2)
Al2—O22	2.113 (3)	Al5—O51	1.767 (2)
Al3—O32	1.710 (2)	Al5—O52	1.786 (2)
Al3—O23	1.796 (2)		

Symmetry code: (i) 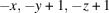.

**Table 2:** Hydrogen-bond geometry (Å, °)

*D*—H⋯*A*	*D*—H	H⋯*A*	*D*⋯*A*	*D*—H⋯*A*
O13—H13⋯O53	0.80 (2)	2.44 (3)	3.080 (3)	138 (4)
O13—H13⋯F15*A*	0.80 (2)	2.63 (4)	3.094 (13)	119 (3)
O13—H13⋯F201	0.80 (2)	2.57 (3)	3.266 (3)	146 (4)
O23—H23⋯F142	0.81 (2)	2.21 (4)	2.876 (4)	139 (5)
O51—H51⋯F53^i^	0.80 (2)	2.07 (2)	2.850 (3)	163 (5)
O52—H52⋯F173^i^	0.81 (2)	2.21 (4)	2.841 (6)	136 (5)
O52—H52⋯F17*A* ^i^	0.81 (2)	2.15 (4)	2.806 (12)	139 (5)
O52—H52⋯F17*B* ^i^	0.81 (2)	2.58 (5)	3.123 (19)	126 (4)
C1—H1*A*⋯O21	1.00	2.48	3.103 (4)	120
C4—H4*A*⋯F81	1.00	2.32	3.023 (5)	126
C4—H4*A*⋯F93	1.00	2.52	3.265 (5)	131
C7—H7*A*⋯O41	1.00	2.59	3.204 (5)	120
C7—H7*A*⋯F183	1.00	2.43	3.336 (6)	151
C10—H10*A*⋯O41	1.00	2.19	2.910 (5)	127
C13*A*—H13*A*⋯F51^ii^	1.00	2.32	3.171 (5)	142
C13*B*—H13*B*⋯O23	1.00	2.51	3.090 (14)	116
C16*B*—H16*B*⋯F12*A*	1.00	2.19	2.969 (18)	133

Symmetry codes: (i) 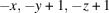; (ii) 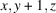.

**Table 3:** Experimental details

Crystal data	
Chemical formula	[Al_10_(C_3_HF_6_O)_14_(OH)_8_O_4_]
*M*_r_	2808.39
Crystal system, space group	Triclinic, *P* 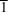
Temperature (K)	100
*a*, *b*, *c* (Å)	11.8721 (8), 12.4448 (8), 16.3091 (11)
α, β, γ (°)	108.754 (3), 102.232 (3), 98.650 (3)
*V* (Å^3^)	2166.8 (3)
*Z*	1
Radiation type	Mo *K*α
μ (mm^−1^)	0.37
Crystal size (mm)	0.20 × 0.20 × 0.20

Data collection	
Diffractometer	Bruker APEXII CCD
Absorption correction	Multi-scan (*SADABS*; Bruker, 2016 ▸)
*T*_min_, *T*_max_	0.634, 0.747
No. of measured, independent and observed [*I* > 2σ(*I*)] reflections	13173, 13173, 8076
*R*_int_	0.075
(sin θ/λ)_max_ (Å^−1^)	0.714

Refinement	
*R*[*F* ^2^ > 2σ(*F* ^2^)], *wR*(*F* ^2^), *S*	0.059, 0.171, 1.02
No. of reflections	13173
No. of parameters	935
No. of restraints	307
H-atom treatment	H atoms treated by a mixture of independent and constrained refinement
Δρ_max_, Δρ_min_ (e Å^−3^)	0.79, −0.87

Computer programs: *APEX2* and *SAINT* (Bruker, 2016 ▸), *SHELXT* (Sheldrick 2015*a* ▸), *SHELXL2018/3* (Sheldrick, 2015*b* ▸) and *SHELXTL* (Sheldrick 2008 ▸).

## supplementary crystallographic information

### Crystal data

**Table:**

[Al_10_(C_3_HF_6_O)_14_(OH)_8_O_4_]	*Z* = 1
*M**_r_* = 2808.39	*F*(000) = 1368
Triclinic, *P*1	*D*_x_ = 2.152 Mg m^−^^3^
*a* = 11.8721 (8) Å	Mo *K*α radiation, λ = 0.71073 Å
*b* = 12.4448 (8) Å	Cell parameters from 8011 reflections
*c* = 16.3091 (11) Å	θ = 2.6–33.8°
α = 108.754 (3)°	µ = 0.37 mm^−^^1^
β = 102.232 (3)°	*T* = 100 K
γ = 98.650 (3)°	Chunk, colorless
*V* = 2166.8 (3) Å^3^	0.20 × 0.20 × 0.20 mm

### Data collection

**Table:**

Bruker APEXII CCD diffractometer	8076 reflections with *I* > 2σ(*I*)
φ and ω scans	*R*_int_ = 0.075
Absorption correction: multi-scan (SADABS; Bruker, 2016)	θ_max_ = 30.5°, θ_min_ = 2.6°
*T*_min_ = 0.634, *T*_max_ = 0.747	*h* = −16→16
13173 measured reflections	*k* = −17→17
13173 independent reflections	*l* = 0→23

### Refinement

**Table:**

Refinement on *F*^2^	Primary atom site location: structure-invariant direct methods
Least-squares matrix: full	Secondary atom site location: difference Fourier map
*R*[*F*^2^ > 2σ(*F*^2^)] = 0.059	Hydrogen site location: mixed
*wR*(*F*^2^) = 0.171	H atoms treated by a mixture of independent and constrained refinement
*S* = 1.02	*w* = 1/[σ^2^(*F*_o_^2^) + (0.0742*P*)^2^ + 2.7605*P*] where *P* = (*F*_o_^2^ + 2*F*_c_^2^)/3
13173 reflections	(Δ/σ)_max_ = 0.001
935 parameters	Δρ_max_ = 0.79 e Å^−^^3^
307 restraints	Δρ_min_ = −0.87 e Å^−^^3^

### Special details

**Table:**

Geometry. All esds (except the esd in the dihedral angle between two l.s. planes) are estimated using the full covariance matrix. The cell esds are taken into account individually in the estimation of esds in distances, angles and torsion angles; correlations between esds in cell parameters are only used when they are defined by crystal symmetry. An approximate (isotropic) treatment of cell esds is used for estimating esds involving l.s. planes.

### Fractional atomic coordinates and isotropic or equivalent isotropic displacement parameters (Å^2^)

**Table:**

	*x*	*y*	*z*	*U*_iso_*/*U*_eq_	Occ. (<1)
Al1	0.11057 (7)	0.56017 (7)	0.55578 (6)	0.01470 (17)	
Al2	0.26603 (8)	0.51056 (7)	0.69630 (6)	0.01955 (19)	
Al3	0.28902 (8)	0.74967 (7)	0.69685 (6)	0.01924 (18)	
Al4	0.12747 (9)	0.66333 (8)	0.78809 (6)	0.02044 (19)	
Al5	0.01488 (8)	0.58474 (7)	0.35584 (6)	0.01725 (18)	
O1	0.16224 (18)	0.60890 (16)	0.68017 (13)	0.0165 (4)	
O11	0.02188 (18)	0.54022 (16)	0.44686 (13)	0.0159 (4)	
O12	0.22217 (18)	0.47319 (16)	0.56169 (14)	0.0177 (4)	
O13	0.20279 (19)	0.70523 (17)	0.58119 (14)	0.0196 (4)	
H13	0.207 (4)	0.740 (3)	0.548 (2)	0.029 (11)*	
O21	0.3669 (2)	0.42397 (19)	0.69781 (17)	0.0288 (5)	
O22	0.2458 (2)	0.59639 (19)	0.82515 (15)	0.0263 (5)	
O23	0.37364 (19)	0.64705 (18)	0.71473 (15)	0.0221 (4)	
H23	0.444 (2)	0.655 (5)	0.720 (4)	0.077 (19)*	
O31	0.2105 (2)	0.80585 (17)	0.78288 (14)	0.0231 (4)	
O32	0.3947 (2)	0.87142 (19)	0.71596 (17)	0.0274 (5)	
O41	0.0835 (2)	0.7390 (2)	0.88066 (15)	0.0296 (5)	
O51	−0.12965 (19)	0.60400 (17)	0.32113 (14)	0.0204 (4)	
H51	−0.147 (4)	0.663 (3)	0.320 (3)	0.046 (13)*	
O52	0.0089 (2)	0.45898 (18)	0.26165 (14)	0.0209 (4)	
H52	0.071 (3)	0.453 (4)	0.250 (3)	0.050 (15)*	
O53	0.13129 (19)	0.70295 (18)	0.38762 (15)	0.0230 (4)	
C1	0.2654 (3)	0.3974 (2)	0.4988 (2)	0.0231 (6)	
H1A	0.330262	0.372176	0.532717	0.028*	
C2	0.3190 (3)	0.4619 (3)	0.4455 (2)	0.0256 (6)	
C3	0.1678 (3)	0.2884 (3)	0.4387 (2)	0.0255 (6)	
C4	0.4216 (3)	0.3511 (3)	0.7308 (3)	0.0308 (7)	
H4A	0.423231	0.371894	0.795689	0.037*	
C5	0.3526 (4)	0.2238 (3)	0.6787 (4)	0.0480 (12)	
C6	0.5489 (4)	0.3677 (4)	0.7243 (3)	0.0458 (10)	
C7	0.2915 (3)	0.6084 (3)	0.9154 (2)	0.0321 (8)	
H7A	0.263162	0.673363	0.953491	0.039*	
C8	0.4268 (4)	0.6439 (3)	0.9444 (3)	0.0401 (9)	
C9	0.2392 (4)	0.4976 (3)	0.9286 (3)	0.0386 (9)	
C10	0.2067 (4)	0.9202 (3)	0.8327 (3)	0.0417 (10)	
H10A	0.151426	0.905456	0.868472	0.050*	
C11	0.1378 (3)	0.9727 (3)	0.7752 (2)	0.0334 (8)	
C12	0.3130 (5)	0.9939 (4)	0.9039 (3)	0.0632 (16)	
C13A	0.4999 (4)	0.9295 (4)	0.7147 (3)	0.0244 (10)	0.769 (9)
H13A	0.501453	1.014669	0.738237	0.029*	0.769 (9)
C13B	0.5064 (11)	0.8590 (11)	0.6855 (8)	0.027 (3)	0.231 (9)
H13B	0.503315	0.773577	0.661533	0.032*	0.231 (9)
C14	0.6050 (3)	0.9124 (4)	0.7739 (3)	0.0396 (9)	
C15	0.5077 (3)	0.8973 (3)	0.6158 (3)	0.0358 (8)	
C16A	−0.0067 (4)	0.7160 (4)	0.9209 (3)	0.0353 (12)	0.810 (10)
H16A	−0.026917	0.630059	0.907757	0.042*	0.810 (10)
C16B	0.0146 (12)	0.7868 (14)	0.9287 (8)	0.036 (3)	0.190 (10)
H16B	0.036292	0.872113	0.939764	0.044*	0.190 (10)
C17	−0.1147 (4)	0.7478 (4)	0.8830 (3)	0.0519 (11)	
C18	0.0456 (5)	0.7778 (5)	1.0222 (3)	0.0555 (12)	
C19	0.1634 (3)	0.8018 (3)	0.3681 (2)	0.0264 (7)	
H19A	0.251891	0.823834	0.383337	0.032*	
C20	0.1233 (3)	0.9019 (3)	0.4273 (3)	0.0349 (8)	
C21	0.1143 (4)	0.7783 (4)	0.2689 (3)	0.0395 (9)	
F21	0.40343 (18)	0.55535 (17)	0.50202 (15)	0.0329 (5)	
F22	0.36696 (19)	0.39535 (19)	0.38769 (15)	0.0367 (5)	
F23	0.23906 (17)	0.50107 (18)	0.39799 (15)	0.0319 (4)	
F31	0.1236 (2)	0.23738 (16)	0.48875 (14)	0.0337 (5)	
F32	0.2074 (2)	0.21197 (16)	0.38103 (15)	0.0357 (5)	
F33	0.07529 (18)	0.31398 (15)	0.38976 (14)	0.0305 (4)	
F51	0.4002 (3)	0.1510 (2)	0.7111 (3)	0.0794 (11)	
F52	0.3431 (3)	0.1894 (2)	0.5914 (2)	0.0674 (9)	
F53	0.2415 (2)	0.2112 (2)	0.6876 (2)	0.0563 (8)	
F61	0.5550 (3)	0.3428 (3)	0.6405 (2)	0.0734 (9)	
F62	0.6065 (3)	0.3021 (3)	0.7593 (3)	0.0883 (12)	
F63	0.6070 (2)	0.4787 (3)	0.7698 (2)	0.0617 (8)	
F81	0.4754 (2)	0.5590 (2)	0.9043 (2)	0.0604 (8)	
F82	0.4682 (3)	0.6717 (3)	1.03286 (17)	0.0633 (8)	
F83	0.4654 (2)	0.7349 (2)	0.92514 (19)	0.0592 (8)	
F91	0.2797 (3)	0.5033 (3)	1.01232 (17)	0.0639 (8)	
F92	0.1210 (2)	0.4799 (2)	0.90928 (17)	0.0466 (6)	
F93	0.2623 (2)	0.40254 (19)	0.87329 (17)	0.0462 (6)	
F111	0.0246 (3)	0.9176 (3)	0.7390 (3)	0.0423 (10)	0.766 (8)
F112	0.1833 (4)	0.9652 (3)	0.7039 (2)	0.0365 (9)	0.766 (8)
F113	0.1425 (13)	1.0850 (5)	0.8181 (8)	0.0471 (19)	0.766 (8)
F11A	0.1112 (13)	0.9412 (10)	0.6913 (6)	0.039 (2)	0.234 (8)
F11B	0.0339 (9)	0.9206 (9)	0.7929 (9)	0.043 (3)	0.234 (8)
F11C	0.152 (4)	1.0857 (15)	0.818 (2)	0.043 (5)	0.234 (8)
F121	0.3881 (4)	0.9365 (4)	0.9299 (3)	0.0510 (12)	0.747 (5)
F122	0.3832 (3)	1.0585 (2)	0.8632 (2)	0.0426 (9)	0.747 (5)
F123	0.2992 (3)	1.0761 (3)	0.9702 (2)	0.0420 (9)	0.747 (5)
F12A	0.2041 (9)	0.9938 (9)	0.9627 (6)	0.058 (3)	0.253 (5)
F12B	0.3562 (11)	1.0866 (8)	0.9515 (8)	0.059 (3)	0.253 (5)
F12C	0.3444 (12)	0.9107 (10)	0.9488 (8)	0.054 (4)	0.253 (5)
F141	0.6026 (4)	0.9477 (4)	0.8585 (2)	0.0577 (11)	0.772 (5)
F142	0.6042 (2)	0.7947 (2)	0.7535 (2)	0.0445 (9)	0.772 (5)
F143	0.7090 (4)	0.9608 (4)	0.7700 (5)	0.0487 (12)	0.772 (5)
F14A	0.6003 (10)	1.0389 (9)	0.8026 (8)	0.064 (3)	0.228 (5)
F14B	0.7117 (13)	0.9269 (14)	0.7610 (16)	0.047 (3)	0.228 (5)
F14C	0.5929 (14)	0.8838 (14)	0.8368 (9)	0.067 (3)	0.228 (5)
F151	0.5990 (4)	0.9655 (4)	0.6102 (3)	0.0625 (12)	0.771 (4)
F152	0.5152 (3)	0.7868 (3)	0.5815 (2)	0.0520 (9)	0.771 (4)
F153	0.4089 (3)	0.9058 (4)	0.5657 (2)	0.0555 (10)	0.771 (4)
F15A	0.4180 (10)	0.8263 (12)	0.5382 (8)	0.063 (3)	0.229 (4)
F15B	0.5025 (12)	1.0082 (9)	0.6226 (8)	0.059 (3)	0.229 (4)
F15C	0.6027 (10)	0.8800 (13)	0.5804 (9)	0.065 (3)	0.229 (4)
F171	−0.0945 (4)	0.8644 (3)	0.8931 (3)	0.0589 (11)	0.780 (6)
F172	−0.2023 (5)	0.7318 (5)	0.9199 (5)	0.0695 (14)	0.780 (6)
F173	−0.1589 (5)	0.6903 (4)	0.7952 (3)	0.0588 (13)	0.780 (6)
F17A	−0.1343 (12)	0.6147 (9)	0.8658 (9)	0.070 (3)	0.220 (6)
F17B	−0.1457 (19)	0.7353 (15)	0.7975 (9)	0.060 (4)	0.220 (6)
F17C	−0.1910 (18)	0.7726 (16)	0.9252 (16)	0.070 (4)	0.220 (6)
F181	0.0757 (5)	0.8906 (3)	1.0466 (2)	0.0714 (14)	0.759 (5)
F182	−0.0366 (5)	0.7525 (4)	1.0639 (3)	0.0745 (13)	0.759 (5)
F183	0.1407 (5)	0.7423 (5)	1.0517 (2)	0.0733 (14)	0.759 (5)
F18A	0.0476 (15)	0.6767 (10)	1.0247 (8)	0.075 (3)	0.241 (5)
F18B	0.1690 (10)	0.8383 (13)	1.0602 (7)	0.074 (3)	0.241 (5)
F18C	−0.0039 (13)	0.8409 (14)	1.0782 (8)	0.083 (3)	0.241 (5)
F201	0.1731 (3)	0.9221 (2)	0.51341 (17)	0.0568 (7)	
F202	0.1527 (3)	0.9997 (2)	0.4134 (2)	0.0650 (8)	
F203	0.0058 (2)	0.8768 (2)	0.41380 (19)	0.0481 (6)	
F211	0.1522 (3)	0.8649 (3)	0.2456 (2)	0.0821 (12)	
F212	0.1437 (3)	0.6820 (3)	0.21900 (19)	0.0652 (8)	
F213	−0.0046 (2)	0.7514 (2)	0.24250 (16)	0.0463 (6)	

### Atomic displacement parameters (Å^2^)

**Table:**

	*U*^11^	*U*^22^	*U*^33^	*U*^12^	*U*^13^	*U*^23^
Al1	0.0170 (4)	0.0096 (3)	0.0159 (4)	0.0015 (3)	0.0003 (3)	0.0060 (3)
Al2	0.0203 (4)	0.0125 (4)	0.0230 (4)	0.0013 (3)	−0.0022 (3)	0.0093 (3)
Al3	0.0207 (4)	0.0117 (4)	0.0216 (4)	−0.0001 (3)	−0.0003 (3)	0.0069 (3)
Al4	0.0275 (5)	0.0156 (4)	0.0145 (4)	0.0014 (3)	0.0014 (4)	0.0051 (3)
Al5	0.0210 (4)	0.0127 (4)	0.0172 (4)	0.0010 (3)	0.0013 (3)	0.0083 (3)
O1	0.0198 (10)	0.0100 (8)	0.0170 (9)	0.0007 (7)	0.0000 (8)	0.0059 (7)
O11	0.0188 (9)	0.0111 (8)	0.0167 (9)	0.0012 (7)	0.0005 (8)	0.0073 (7)
O12	0.0177 (9)	0.0116 (8)	0.0218 (10)	0.0035 (7)	0.0022 (8)	0.0057 (7)
O13	0.0241 (10)	0.0125 (9)	0.0195 (10)	−0.0003 (8)	−0.0011 (8)	0.0090 (8)
O21	0.0270 (12)	0.0199 (10)	0.0383 (13)	0.0062 (9)	−0.0032 (10)	0.0167 (10)
O22	0.0307 (12)	0.0223 (10)	0.0191 (10)	0.0014 (9)	−0.0055 (9)	0.0089 (8)
O23	0.0172 (10)	0.0152 (9)	0.0296 (11)	−0.0006 (8)	−0.0020 (9)	0.0098 (8)
O31	0.0295 (11)	0.0119 (9)	0.0207 (10)	0.0022 (8)	−0.0001 (9)	0.0023 (8)
O32	0.0208 (11)	0.0172 (10)	0.0408 (13)	−0.0013 (8)	0.0041 (10)	0.0117 (9)
O41	0.0401 (14)	0.0258 (11)	0.0199 (11)	0.0020 (10)	0.0104 (10)	0.0058 (9)
O51	0.0231 (10)	0.0142 (9)	0.0234 (10)	0.0030 (8)	−0.0010 (8)	0.0115 (8)
O52	0.0251 (11)	0.0182 (10)	0.0167 (10)	0.0010 (8)	0.0034 (9)	0.0062 (8)
O53	0.0238 (11)	0.0181 (10)	0.0271 (11)	0.0003 (8)	0.0023 (9)	0.0135 (9)
C1	0.0243 (15)	0.0144 (12)	0.0288 (15)	0.0093 (11)	0.0038 (12)	0.0057 (11)
C2	0.0211 (14)	0.0237 (14)	0.0310 (16)	0.0090 (12)	0.0069 (13)	0.0071 (12)
C3	0.0317 (16)	0.0141 (12)	0.0288 (16)	0.0088 (12)	0.0050 (13)	0.0059 (11)
C4	0.0289 (17)	0.0284 (16)	0.0386 (19)	0.0113 (13)	0.0019 (15)	0.0196 (14)
C5	0.038 (2)	0.0271 (18)	0.081 (3)	0.0148 (16)	0.004 (2)	0.028 (2)
C6	0.031 (2)	0.045 (2)	0.067 (3)	0.0109 (17)	0.008 (2)	0.029 (2)
C7	0.040 (2)	0.0300 (16)	0.0182 (14)	0.0025 (14)	−0.0054 (14)	0.0099 (13)
C8	0.045 (2)	0.0339 (19)	0.0279 (18)	0.0007 (16)	−0.0131 (16)	0.0121 (15)
C9	0.053 (2)	0.0344 (19)	0.0276 (17)	0.0073 (17)	0.0009 (17)	0.0187 (15)
C10	0.055 (2)	0.0179 (15)	0.0345 (19)	0.0089 (15)	−0.0085 (18)	0.0005 (13)
C11	0.044 (2)	0.0183 (14)	0.0336 (18)	0.0120 (14)	0.0073 (16)	0.0047 (13)
C12	0.079 (4)	0.030 (2)	0.040 (2)	0.017 (2)	−0.020 (2)	−0.0185 (18)
C13A	0.0237 (19)	0.0166 (18)	0.030 (2)	0.0003 (15)	0.0039 (16)	0.0089 (16)
C13B	0.023 (4)	0.019 (4)	0.030 (4)	0.000 (4)	0.008 (4)	0.000 (4)
C14	0.0275 (18)	0.042 (2)	0.040 (2)	−0.0022 (15)	0.0032 (16)	0.0121 (17)
C15	0.0317 (18)	0.0323 (18)	0.040 (2)	0.0052 (14)	0.0080 (16)	0.0118 (15)
C16A	0.053 (3)	0.029 (2)	0.030 (2)	0.010 (2)	0.023 (2)	0.0108 (18)
C16B	0.051 (5)	0.030 (5)	0.029 (5)	0.007 (5)	0.021 (5)	0.006 (5)
C17	0.052 (3)	0.055 (3)	0.047 (3)	0.006 (2)	0.027 (2)	0.010 (2)
C18	0.075 (3)	0.068 (3)	0.032 (2)	0.026 (3)	0.027 (2)	0.018 (2)
C19	0.0239 (15)	0.0234 (15)	0.0332 (17)	−0.0025 (12)	0.0031 (13)	0.0187 (13)
C20	0.0354 (19)	0.0185 (15)	0.051 (2)	0.0002 (13)	0.0064 (17)	0.0188 (15)
C21	0.038 (2)	0.041 (2)	0.040 (2)	−0.0048 (16)	0.0076 (17)	0.0237 (17)
F21	0.0254 (10)	0.0266 (10)	0.0419 (12)	0.0011 (8)	0.0096 (9)	0.0082 (9)
F22	0.0319 (11)	0.0382 (12)	0.0408 (12)	0.0153 (9)	0.0169 (10)	0.0080 (9)
F23	0.0251 (10)	0.0386 (11)	0.0434 (12)	0.0111 (8)	0.0112 (9)	0.0272 (10)
F31	0.0440 (12)	0.0156 (8)	0.0384 (11)	−0.0006 (8)	0.0105 (10)	0.0098 (8)
F32	0.0461 (13)	0.0171 (9)	0.0404 (12)	0.0142 (9)	0.0138 (10)	0.0018 (8)
F33	0.0309 (10)	0.0160 (8)	0.0338 (10)	0.0053 (7)	−0.0021 (8)	0.0023 (7)
F51	0.0578 (18)	0.0412 (15)	0.151 (3)	0.0253 (13)	0.0084 (19)	0.0574 (19)
F52	0.0657 (19)	0.0363 (14)	0.076 (2)	0.0146 (13)	0.0066 (16)	−0.0034 (13)
F53	0.0365 (13)	0.0308 (12)	0.109 (2)	0.0076 (10)	0.0110 (14)	0.0413 (14)
F61	0.066 (2)	0.076 (2)	0.082 (2)	0.0088 (16)	0.0419 (18)	0.0229 (18)
F62	0.0382 (15)	0.095 (3)	0.163 (4)	0.0369 (17)	0.0193 (19)	0.083 (3)
F63	0.0311 (13)	0.0567 (17)	0.087 (2)	−0.0011 (11)	0.0014 (13)	0.0272 (15)
F81	0.0388 (14)	0.0522 (16)	0.0658 (18)	0.0068 (12)	−0.0079 (13)	0.0057 (13)
F82	0.0639 (18)	0.0698 (18)	0.0297 (12)	−0.0006 (14)	−0.0236 (12)	0.0143 (12)
F83	0.0455 (15)	0.0531 (15)	0.0628 (17)	−0.0143 (12)	−0.0207 (13)	0.0344 (14)
F91	0.090 (2)	0.0623 (17)	0.0378 (13)	0.0019 (15)	−0.0044 (14)	0.0369 (13)
F92	0.0523 (15)	0.0454 (14)	0.0475 (14)	0.0052 (11)	0.0123 (12)	0.0277 (11)
F93	0.0594 (15)	0.0288 (11)	0.0516 (14)	0.0073 (10)	0.0068 (12)	0.0233 (10)
F111	0.0338 (16)	0.0329 (15)	0.047 (2)	0.0089 (12)	−0.0044 (15)	0.0075 (15)
F112	0.054 (2)	0.0290 (15)	0.0305 (15)	0.0144 (16)	0.0087 (16)	0.0164 (12)
F113	0.066 (4)	0.019 (2)	0.051 (3)	0.019 (2)	0.010 (3)	0.005 (2)
F11A	0.049 (5)	0.040 (4)	0.031 (4)	0.015 (4)	0.009 (4)	0.015 (3)
F11B	0.040 (5)	0.045 (5)	0.044 (6)	0.020 (4)	0.004 (5)	0.016 (5)
F11C	0.060 (8)	0.019 (7)	0.045 (7)	0.010 (7)	0.020 (7)	0.003 (7)
F121	0.050 (3)	0.039 (2)	0.040 (2)	0.0067 (19)	−0.0146 (18)	0.0026 (15)
F122	0.0452 (18)	0.0242 (14)	0.0422 (17)	−0.0091 (12)	0.0133 (14)	−0.0018 (12)
F123	0.049 (2)	0.0297 (15)	0.0265 (15)	−0.0033 (15)	0.0084 (14)	−0.0091 (12)
F12A	0.074 (6)	0.042 (5)	0.037 (5)	−0.003 (5)	0.014 (5)	−0.001 (4)
F12B	0.062 (5)	0.033 (4)	0.047 (4)	−0.018 (4)	0.001 (4)	−0.007 (4)
F12C	0.064 (7)	0.032 (5)	0.033 (6)	0.010 (5)	−0.024 (5)	−0.006 (4)
F141	0.057 (2)	0.076 (3)	0.0361 (19)	0.021 (2)	−0.0002 (17)	0.0209 (19)
F142	0.0278 (14)	0.0340 (15)	0.076 (2)	0.0076 (11)	0.0053 (14)	0.0321 (15)
F143	0.0240 (16)	0.039 (3)	0.082 (3)	−0.0015 (15)	0.0010 (17)	0.033 (2)
F14A	0.038 (5)	0.061 (5)	0.064 (6)	0.002 (4)	0.002 (4)	−0.002 (5)
F14B	0.026 (5)	0.045 (7)	0.074 (6)	0.008 (5)	0.012 (4)	0.029 (6)
F14C	0.060 (5)	0.073 (6)	0.060 (6)	−0.006 (6)	−0.006 (5)	0.040 (5)
F151	0.062 (2)	0.070 (3)	0.056 (2)	−0.014 (2)	0.0215 (18)	0.034 (2)
F152	0.068 (2)	0.0399 (17)	0.0443 (18)	0.0142 (16)	0.0217 (17)	0.0052 (14)
F153	0.052 (2)	0.080 (3)	0.0407 (19)	0.0211 (19)	0.0042 (16)	0.0322 (19)
F15A	0.057 (5)	0.070 (6)	0.043 (5)	0.004 (5)	0.014 (4)	0.002 (5)
F15B	0.071 (6)	0.060 (5)	0.061 (5)	0.012 (5)	0.023 (5)	0.043 (4)
F15C	0.061 (5)	0.074 (6)	0.064 (5)	0.018 (5)	0.029 (5)	0.021 (5)
F171	0.080 (3)	0.0401 (18)	0.064 (2)	0.0270 (17)	0.0220 (19)	0.0209 (16)
F172	0.068 (3)	0.065 (3)	0.087 (3)	0.026 (3)	0.052 (2)	0.019 (3)
F173	0.054 (2)	0.064 (3)	0.044 (2)	0.027 (2)	0.0108 (17)	−0.0024 (19)
F17A	0.065 (6)	0.076 (6)	0.060 (6)	0.012 (5)	0.035 (5)	0.007 (5)
F17B	0.064 (6)	0.057 (7)	0.053 (6)	0.031 (6)	0.009 (5)	0.009 (5)
F17C	0.069 (6)	0.060 (7)	0.078 (6)	0.026 (6)	0.044 (5)	0.002 (6)
F181	0.114 (4)	0.046 (2)	0.0299 (17)	0.009 (2)	0.009 (2)	−0.0055 (15)
F182	0.120 (4)	0.084 (3)	0.046 (2)	0.035 (3)	0.056 (2)	0.033 (2)
F183	0.099 (3)	0.101 (4)	0.0293 (18)	0.048 (3)	0.020 (2)	0.024 (2)
F18A	0.113 (7)	0.081 (6)	0.048 (5)	0.022 (5)	0.031 (5)	0.042 (5)
F18B	0.100 (6)	0.083 (6)	0.025 (4)	0.018 (6)	0.008 (5)	0.007 (5)
F18C	0.112 (6)	0.088 (6)	0.045 (5)	0.023 (6)	0.044 (5)	0.006 (5)
F201	0.0741 (19)	0.0386 (13)	0.0399 (14)	0.0095 (13)	0.0019 (13)	0.0019 (11)
F202	0.0695 (18)	0.0232 (11)	0.109 (2)	0.0059 (11)	0.0221 (17)	0.0371 (14)
F203	0.0374 (13)	0.0342 (12)	0.0728 (18)	0.0107 (10)	0.0176 (12)	0.0173 (12)
F211	0.091 (2)	0.081 (2)	0.0581 (17)	−0.0438 (18)	−0.0124 (16)	0.0544 (17)
F212	0.0686 (19)	0.090 (2)	0.0387 (14)	0.0245 (17)	0.0246 (14)	0.0176 (15)
F213	0.0375 (12)	0.0553 (15)	0.0442 (13)	−0.0011 (11)	−0.0031 (10)	0.0305 (12)

### Geometric parameters (Å, º)

**Table:**

Al1—O11	1.781 (2)	C9—F92	1.340 (5)
Al1—O13	1.833 (2)	C9—F93	1.343 (5)
Al1—O12	1.839 (2)	C10—C12	1.459 (6)
Al1—O11^i^	1.839 (2)	C10—C11	1.483 (5)
Al1—O1	1.852 (2)	C10—H10A	1.0000
Al2—O21	1.729 (2)	C11—F11A	1.250 (9)
Al2—O23	1.861 (2)	C11—F11C	1.320 (17)
Al2—O51^i^	1.893 (2)	C11—F111	1.324 (5)
Al2—O1	1.900 (2)	C11—F113	1.333 (7)
Al2—O12	2.023 (2)	C11—F112	1.365 (5)
Al2—O22	2.113 (3)	C11—F11B	1.434 (10)
Al3—O32	1.710 (2)	C12—F12B	1.129 (9)
Al3—O23	1.796 (2)	C12—F123	1.287 (5)
Al3—O13	1.803 (2)	C12—F121	1.309 (6)
Al3—O31	1.856 (3)	C12—F122	1.467 (7)
Al3—O1	2.034 (2)	C12—F12C	1.495 (11)
Al4—O41	1.734 (3)	C12—F12A	1.766 (10)
Al4—O22	1.830 (2)	C13A—C14	1.496 (6)
Al4—O1	1.831 (2)	C13A—C15	1.559 (6)
Al4—O52^i^	1.872 (2)	C13A—H13A	1.0000
Al4—O31	1.932 (2)	C13B—C15	1.369 (14)
Al5—O53	1.714 (2)	C13B—C14	1.524 (12)
Al5—O11	1.734 (2)	C13B—H13B	1.0000
Al5—O51	1.767 (2)	C14—F14C	1.216 (11)
Al5—O52	1.786 (2)	C14—F143	1.314 (6)
O12—C1	1.397 (4)	C14—F141	1.314 (5)
O13—H13	0.798 (19)	C14—F14B	1.323 (13)
O21—C4	1.371 (4)	C14—F142	1.391 (5)
O22—C7	1.407 (4)	C14—F14A	1.505 (10)
O23—H23	0.81 (2)	C15—F151	1.311 (5)
O31—C10	1.407 (4)	C15—F153	1.319 (5)
O32—C13A	1.353 (5)	C15—F152	1.332 (5)
O32—C13B	1.523 (14)	C15—F15B	1.361 (10)
O41—C16B	1.325 (16)	C15—F15C	1.385 (11)
O41—C16A	1.409 (5)	C15—F15A	1.394 (10)
O51—H51	0.804 (19)	C16A—C17	1.469 (7)
O52—H52	0.807 (19)	C16A—C18	1.520 (6)
O53—C19	1.386 (3)	C16A—H16A	1.0000
C1—C3	1.528 (4)	C16B—C17	1.485 (14)
C1—C2	1.529 (5)	C16B—C18	1.535 (13)
C1—H1A	1.0000	C16B—H16B	1.0000
C2—F22	1.329 (4)	C17—F17C	1.265 (13)
C2—F21	1.332 (4)	C17—F17B	1.316 (13)
C2—F23	1.345 (4)	C17—F173	1.323 (6)
C3—F31	1.326 (4)	C17—F172	1.327 (7)
C3—F32	1.326 (4)	C17—F171	1.383 (6)
C3—F33	1.357 (4)	C17—F17A	1.561 (11)
C4—C6	1.524 (6)	C18—F18A	1.276 (11)
C4—C5	1.534 (6)	C18—F181	1.298 (6)
C4—H4A	1.0000	C18—F18C	1.305 (10)
C5—F52	1.322 (6)	C18—F183	1.327 (6)
C5—F51	1.325 (4)	C18—F182	1.357 (6)
C5—F53	1.348 (5)	C18—F18B	1.443 (12)
C6—F61	1.321 (6)	C19—C21	1.513 (5)
C6—F62	1.328 (5)	C19—C20	1.521 (5)
C6—F63	1.331 (5)	C19—H19A	1.0000
C7—C9	1.524 (5)	C20—F202	1.318 (4)
C7—C8	1.526 (6)	C20—F201	1.327 (5)
C7—H7A	1.0000	C20—F203	1.336 (5)
C8—F83	1.314 (5)	C21—F211	1.303 (4)
C8—F82	1.330 (4)	C21—F213	1.340 (5)
C8—F81	1.332 (5)	C21—F212	1.357 (5)
C9—F91	1.322 (4)		
			
O11—Al1—O13	97.78 (9)	F82—C8—F81	107.2 (3)
O11—Al1—O12	117.46 (10)	F83—C8—C7	110.9 (3)
O13—Al1—O12	101.90 (10)	F82—C8—C7	110.5 (4)
O11—Al1—O11^i^	81.79 (10)	F81—C8—C7	112.7 (3)
O13—Al1—O11^i^	153.71 (11)	F91—C9—F92	108.0 (4)
O12—Al1—O11^i^	101.50 (9)	F91—C9—F93	108.0 (3)
O11—Al1—O1	159.53 (10)	F92—C9—F93	106.6 (3)
O13—Al1—O1	80.54 (9)	F91—C9—C7	112.3 (3)
O12—Al1—O1	82.64 (9)	F92—C9—C7	109.9 (3)
O11^i^—Al1—O1	90.78 (9)	F93—C9—C7	111.9 (3)
O21—Al2—O23	96.86 (11)	O31—C10—C12	117.6 (3)
O21—Al2—O51^i^	97.40 (11)	O31—C10—C11	111.8 (3)
O23—Al2—O51^i^	165.72 (11)	C12—C10—C11	119.0 (3)
O21—Al2—O1	171.59 (12)	O31—C10—H10A	101.5
O23—Al2—O1	78.91 (9)	C12—C10—H10A	101.5
O51^i^—Al2—O1	87.04 (9)	C11—C10—H10A	101.5
O21—Al2—O12	96.15 (11)	F11A—C11—F11C	114.2 (18)
O23—Al2—O12	90.33 (10)	F111—C11—F113	107.6 (7)
O51^i^—Al2—O12	88.92 (9)	F111—C11—F112	105.2 (3)
O1—Al2—O12	76.73 (9)	F113—C11—F112	107.5 (5)
O21—Al2—O22	113.06 (11)	F11A—C11—F11B	104.3 (7)
O23—Al2—O22	87.22 (10)	F11C—C11—F11B	103.8 (15)
O51^i^—Al2—O22	86.50 (10)	F11A—C11—C10	127.8 (6)
O1—Al2—O22	74.22 (9)	F11C—C11—C10	112 (2)
O12—Al2—O22	150.78 (9)	F111—C11—C10	114.1 (4)
O32—Al3—O23	103.74 (11)	F113—C11—C10	113.4 (7)
O32—Al3—O13	105.59 (12)	F112—C11—C10	108.6 (3)
O23—Al3—O13	112.56 (11)	F11B—C11—C10	87.5 (6)
O32—Al3—O31	101.95 (11)	F123—C12—F121	112.8 (4)
O23—Al3—O31	115.50 (11)	F12B—C12—C10	143.5 (8)
O13—Al3—O31	115.55 (11)	F123—C12—C10	117.5 (5)
O32—Al3—O1	177.19 (12)	F121—C12—C10	114.3 (4)
O23—Al3—O1	76.95 (9)	F123—C12—F122	102.7 (4)
O13—Al3—O1	76.49 (9)	F121—C12—F122	100.2 (5)
O31—Al3—O1	75.36 (9)	C10—C12—F122	106.8 (4)
O41—Al4—O22	108.96 (12)	F12B—C12—F12C	109.9 (8)
O41—Al4—O1	166.52 (11)	C10—C12—F12C	100.5 (6)
O22—Al4—O1	83.04 (10)	F12B—C12—F12A	88.4 (8)
O41—Al4—O52^i^	95.13 (12)	C10—C12—F12A	77.4 (5)
O22—Al4—O52^i^	104.38 (11)	F12C—C12—F12A	79.9 (7)
O1—Al4—O52^i^	87.50 (10)	O32—C13A—C14	113.7 (3)
O41—Al4—O31	92.28 (11)	O32—C13A—C15	110.1 (3)
O22—Al4—O31	104.14 (11)	C14—C13A—C15	111.6 (3)
O1—Al4—O31	78.49 (9)	O32—C13A—H13A	107.1
O52^i^—Al4—O31	146.29 (10)	C14—C13A—H13A	107.1
O53—Al5—O11	107.38 (10)	C15—C13A—H13A	107.1
O53—Al5—O51	117.54 (11)	C15—C13B—O32	111.3 (9)
O11—Al5—O51	107.62 (11)	C15—C13B—C14	121.5 (9)
O53—Al5—O52	117.44 (12)	O32—C13B—C14	103.1 (8)
O11—Al5—O52	106.56 (10)	C15—C13B—H13B	106.7
O51—Al5—O52	99.46 (11)	O32—C13B—H13B	106.7
Al4—O1—Al1	147.74 (13)	C14—C13B—H13B	106.7
Al4—O1—Al2	104.61 (10)	F143—C14—F141	108.8 (4)
Al1—O1—Al2	102.00 (10)	F14C—C14—F14B	118.0 (12)
Al4—O1—Al3	99.09 (9)	F143—C14—F142	105.2 (4)
Al1—O1—Al3	95.50 (9)	F141—C14—F142	103.0 (4)
Al2—O1—Al3	96.86 (10)	F143—C14—C13A	115.6 (4)
Al5—O11—Al1	144.27 (13)	F141—C14—C13A	111.4 (4)
Al5—O11—Al1^i^	117.37 (11)	F142—C14—C13A	111.9 (3)
Al1—O11—Al1^i^	98.21 (10)	F14C—C14—F14A	105.2 (10)
C1—O12—Al1	135.25 (19)	F14B—C14—F14A	96.7 (8)
C1—O12—Al2	126.73 (18)	F14C—C14—C13B	118.6 (9)
Al1—O12—Al2	97.93 (10)	F14B—C14—C13B	112.3 (11)
Al3—O13—Al1	104.65 (11)	F14A—C14—C13B	101.4 (7)
Al3—O13—H13	127 (3)	F151—C15—F153	109.7 (4)
Al1—O13—H13	128 (3)	F151—C15—F152	108.8 (4)
C4—O21—Al2	152.0 (3)	F153—C15—F152	106.6 (4)
C7—O22—Al4	123.5 (2)	F15B—C15—C13B	122.6 (8)
C7—O22—Al2	139.7 (2)	F15B—C15—F15C	103.5 (8)
Al4—O22—Al2	96.72 (10)	C13B—C15—F15C	114.0 (9)
Al3—O23—Al2	107.19 (12)	F15B—C15—F15A	104.6 (8)
Al3—O23—H23	125 (4)	C13B—C15—F15A	111.5 (8)
Al2—O23—H23	126 (4)	F15C—C15—F15A	97.3 (8)
C10—O31—Al3	131.5 (3)	F151—C15—C13A	111.9 (3)
C10—O31—Al4	126.5 (3)	F153—C15—C13A	108.7 (3)
Al3—O31—Al4	101.97 (10)	F152—C15—C13A	110.9 (3)
C13A—O32—Al3	154.9 (3)	O41—C16A—C17	111.6 (4)
C13B—O32—Al3	120.0 (5)	O41—C16A—C18	107.1 (4)
C16B—O41—Al4	159.7 (5)	C17—C16A—C18	114.5 (4)
C16A—O41—Al4	136.2 (3)	O41—C16A—H16A	107.8
Al5—O51—Al2^i^	121.60 (11)	C17—C16A—H16A	107.8
Al5—O51—H51	127 (4)	C18—C16A—H16A	107.8
Al2^i^—O51—H51	111 (4)	O41—C16B—C17	115.7 (9)
Al5—O52—Al4^i^	119.51 (13)	O41—C16B—C18	110.7 (10)
Al5—O52—H52	116 (4)	C17—C16B—C18	112.7 (9)
Al4^i^—O52—H52	123 (4)	O41—C16B—H16B	105.6
C19—O53—Al5	140.3 (2)	C17—C16B—H16B	105.6
O12—C1—C3	110.3 (3)	C18—C16B—H16B	105.6
O12—C1—C2	109.8 (2)	F17C—C17—F17B	115.7 (14)
C3—C1—C2	113.0 (3)	F173—C17—F172	106.7 (5)
O12—C1—H1A	107.9	F173—C17—F171	104.7 (5)
C3—C1—H1A	107.9	F172—C17—F171	104.8 (4)
C2—C1—H1A	107.9	F173—C17—C16A	112.7 (4)
F22—C2—F21	108.1 (3)	F172—C17—C16A	114.8 (5)
F22—C2—F23	107.2 (3)	F171—C17—C16A	112.4 (4)
F21—C2—F23	106.6 (3)	F17C—C17—C16B	122.0 (12)
F22—C2—C1	112.3 (3)	F17B—C17—C16B	115.7 (11)
F21—C2—C1	109.7 (3)	F17C—C17—F17A	99.8 (10)
F23—C2—C1	112.8 (3)	F17B—C17—F17A	95.8 (8)
F31—C3—F32	108.9 (2)	C16B—C17—F17A	100.0 (8)
F31—C3—F33	106.2 (3)	F18A—C18—F18C	115.6 (9)
F32—C3—F33	107.3 (3)	F181—C18—F183	108.0 (5)
F31—C3—C1	110.2 (3)	F181—C18—F182	107.7 (4)
F32—C3—C1	111.8 (3)	F183—C18—F182	108.2 (4)
F33—C3—C1	112.2 (2)	F18A—C18—F18B	102.1 (10)
O21—C4—C6	109.5 (3)	F18C—C18—F18B	102.1 (9)
O21—C4—C5	110.2 (3)	F181—C18—C16A	112.9 (4)
C6—C4—C5	110.9 (3)	F183—C18—C16A	111.0 (4)
O21—C4—H4A	108.7	F182—C18—C16A	108.9 (5)
C6—C4—H4A	108.7	F18A—C18—C16B	117.0 (8)
C5—C4—H4A	108.7	F18C—C18—C16B	113.6 (9)
F52—C5—F51	108.9 (4)	F18B—C18—C16B	103.5 (7)
F52—C5—F53	107.1 (4)	O53—C19—C21	111.2 (3)
F51—C5—F53	106.1 (4)	O53—C19—C20	109.3 (3)
F52—C5—C4	113.0 (4)	C21—C19—C20	112.1 (3)
F51—C5—C4	112.0 (4)	O53—C19—H19A	108.0
F53—C5—C4	109.5 (4)	C21—C19—H19A	108.0
F61—C6—F62	108.2 (4)	C20—C19—H19A	108.0
F61—C6—F63	106.7 (4)	F202—C20—F201	107.8 (3)
F62—C6—F63	107.7 (4)	F202—C20—F203	107.6 (3)
F61—C6—C4	112.8 (4)	F201—C20—F203	107.4 (3)
F62—C6—C4	111.3 (4)	F202—C20—C19	112.6 (3)
F63—C6—C4	109.9 (4)	F201—C20—C19	109.5 (3)
O22—C7—C9	109.4 (3)	F203—C20—C19	111.6 (3)
O22—C7—C8	111.2 (3)	F211—C21—F213	108.2 (4)
C9—C7—C8	114.4 (3)	F211—C21—F212	108.5 (4)
O22—C7—H7A	107.2	F213—C21—F212	104.1 (3)
C9—C7—H7A	107.2	F211—C21—C19	113.4 (3)
C8—C7—H7A	107.2	F213—C21—C19	112.4 (3)
F83—C8—F82	108.0 (3)	F212—C21—C19	109.7 (3)
F83—C8—F81	107.2 (4)		
			
O41—Al4—O1—Al1	−51.9 (6)	O22—Al4—O41—C16A	−95.6 (4)
O22—Al4—O1—Al1	154.6 (2)	O1—Al4—O41—C16A	112.3 (5)
O52^i^—Al4—O1—Al1	49.8 (2)	O52^i^—Al4—O41—C16A	11.6 (4)
O31—Al4—O1—Al1	−99.4 (2)	O31—Al4—O41—C16A	158.6 (4)
Al3—Al4—O1—Al1	−115.8 (2)	Al3—Al4—O41—C16A	165.3 (3)
Al2—Al4—O1—Al1	144.6 (3)	Al2—Al4—O41—C16A	−91.6 (4)
O41—Al4—O1—Al2	163.5 (5)	O53—Al5—O51—Al2^i^	−169.35 (13)
O22—Al4—O1—Al2	9.99 (11)	O11—Al5—O51—Al2^i^	−48.08 (16)
O52^i^—Al4—O1—Al2	−94.83 (11)	O52—Al5—O51—Al2^i^	62.78 (16)
O31—Al4—O1—Al2	116.01 (11)	O53—Al5—O52—Al4^i^	−170.17 (12)
Al3—Al4—O1—Al2	99.60 (12)	O11—Al5—O52—Al4^i^	69.46 (15)
O41—Al4—O1—Al3	63.9 (5)	O51—Al5—O52—Al4^i^	−42.23 (15)
O22—Al4—O1—Al3	−89.60 (10)	O11—Al5—O53—C19	−153.5 (3)
O52^i^—Al4—O1—Al3	165.58 (10)	O51—Al5—O53—C19	−32.1 (4)
O31—Al4—O1—Al3	16.42 (9)	O52—Al5—O53—C19	86.6 (4)
Al2—Al4—O1—Al3	−99.60 (12)	Al1—O12—C1—C3	−65.4 (3)
O11—Al1—O1—Al4	17.8 (4)	Al2—O12—C1—C3	110.3 (2)
O13—Al1—O1—Al4	104.7 (2)	Al1—O12—C1—C2	59.7 (4)
O12—Al1—O1—Al4	−151.8 (2)	Al2—O12—C1—C2	−124.6 (2)
O11^i^—Al1—O1—Al4	−50.3 (2)	O12—C1—C2—F22	176.8 (2)
Al1^i^—Al1—O1—Al4	−34.7 (3)	C3—C1—C2—F22	−59.6 (3)
Al3—Al1—O1—Al4	116.7 (2)	O12—C1—C2—F21	56.7 (3)
Al2—Al1—O1—Al4	−145.0 (3)	C3—C1—C2—F21	−179.8 (2)
O11—Al1—O1—Al2	162.9 (2)	O12—C1—C2—F23	−61.9 (3)
O13—Al1—O1—Al2	−110.23 (11)	C3—C1—C2—F23	61.6 (3)
O12—Al1—O1—Al2	−6.79 (10)	O12—C1—C3—F31	−55.7 (3)
O11^i^—Al1—O1—Al2	94.71 (10)	C2—C1—C3—F31	−179.0 (2)
Al1^i^—Al1—O1—Al2	110.31 (10)	O12—C1—C3—F32	−177.0 (2)
Al3—Al1—O1—Al2	−98.23 (11)	C2—C1—C3—F32	59.8 (3)
O11—Al1—O1—Al3	−98.9 (3)	O12—C1—C3—F33	62.4 (4)
O13—Al1—O1—Al3	−12.00 (9)	C2—C1—C3—F33	−60.8 (4)
O12—Al1—O1—Al3	91.44 (9)	Al2—O21—C4—C6	−148.4 (4)
O11^i^—Al1—O1—Al3	−167.06 (9)	Al2—O21—C4—C5	89.3 (6)
Al1^i^—Al1—O1—Al3	−151.47 (7)	O21—C4—C5—F52	59.1 (4)
Al2—Al1—O1—Al3	98.23 (11)	C6—C4—C5—F52	−62.3 (4)
O23—Al2—O1—Al4	−99.15 (11)	O21—C4—C5—F51	−177.4 (4)
O51^i^—Al2—O1—Al4	78.27 (11)	C6—C4—C5—F51	61.2 (5)
O12—Al2—O1—Al4	167.86 (11)	O21—C4—C5—F53	−60.1 (5)
O22—Al2—O1—Al4	−8.92 (10)	C6—C4—C5—F53	178.5 (3)
Al1—Al2—O1—Al4	161.57 (15)	O21—C4—C6—F61	−60.9 (4)
Al3—Al2—O1—Al4	−101.29 (11)	C5—C4—C6—F61	61.0 (4)
O23—Al2—O1—Al1	99.28 (11)	O21—C4—C6—F62	177.3 (4)
O51^i^—Al2—O1—Al1	−83.30 (11)	C5—C4—C6—F62	−60.9 (5)
O12—Al2—O1—Al1	6.29 (9)	O21—C4—C6—F63	58.0 (5)
O22—Al2—O1—Al1	−170.49 (12)	C5—C4—C6—F63	179.9 (4)
Al3—Al2—O1—Al1	97.13 (11)	Al4—O22—C7—C9	101.9 (3)
Al4—Al2—O1—Al1	−161.57 (15)	Al2—O22—C7—C9	−75.5 (4)
O23—Al2—O1—Al3	2.15 (10)	Al4—O22—C7—C8	−130.9 (3)
O51^i^—Al2—O1—Al3	179.56 (10)	Al2—O22—C7—C8	51.8 (4)
O12—Al2—O1—Al3	−90.85 (9)	O22—C7—C8—F83	51.0 (4)
O22—Al2—O1—Al3	92.37 (10)	C9—C7—C8—F83	175.5 (3)
Al1—Al2—O1—Al3	−97.13 (11)	O22—C7—C8—F82	170.8 (3)
Al4—Al2—O1—Al3	101.29 (11)	C9—C7—C8—F82	−64.7 (4)
O53—Al5—O11—Al1	−9.2 (3)	O22—C7—C8—F81	−69.3 (4)
O51—Al5—O11—Al1	−136.6 (2)	C9—C7—C8—F81	55.2 (4)
O52—Al5—O11—Al1	117.4 (2)	O22—C7—C9—F91	179.5 (3)
O53—Al5—O11—Al1^i^	164.95 (12)	C8—C7—C9—F91	54.1 (5)
O51—Al5—O11—Al1^i^	37.52 (15)	O22—C7—C9—F92	−60.3 (4)
O52—Al5—O11—Al1^i^	−68.41 (15)	C8—C7—C9—F92	174.3 (3)
O13—Al1—O11—Al5	21.3 (2)	O22—C7—C9—F93	57.9 (4)
O12—Al1—O11—Al5	−86.5 (2)	C8—C7—C9—F93	−67.6 (4)
O11^i^—Al1—O11—Al5	174.8 (3)	Al3—O31—C10—C12	−71.8 (5)
O1—Al1—O11—Al5	105.1 (3)	Al4—O31—C10—C12	109.4 (5)
Al1^i^—Al1—O11—Al5	174.8 (3)	Al3—O31—C10—C11	71.0 (4)
Al3—Al1—O11—Al5	27.0 (3)	Al4—O31—C10—C11	−107.8 (4)
Al2—Al1—O11—Al5	−106.8 (2)	O31—C10—C11—F11A	−18.8 (10)
O13—Al1—O11—Al1^i^	−153.45 (11)	C12—C10—C11—F11A	123.4 (10)
O12—Al1—O11—Al1^i^	98.75 (11)	O31—C10—C11—F11C	−168.8 (16)
O11^i^—Al1—O11—Al1^i^	0.0	C12—C10—C11—F11C	−26.6 (17)
O1—Al1—O11—Al1^i^	−69.7 (3)	O31—C10—C11—F111	62.9 (5)
Al3—Al1—O11—Al1^i^	−147.76 (9)	C12—C10—C11—F111	−154.8 (5)
Al2—Al1—O11—Al1^i^	78.4 (2)	O31—C10—C11—F113	−173.5 (6)
O11—Al1—O12—C1	6.9 (3)	C12—C10—C11—F113	−31.2 (8)
O13—Al1—O12—C1	−98.5 (3)	O31—C10—C11—F112	−54.1 (5)
O11^i^—Al1—O12—C1	93.5 (3)	C12—C10—C11—F112	88.2 (5)
O1—Al1—O12—C1	−177.2 (3)	O31—C10—C11—F11B	87.3 (6)
Al1^i^—Al1—O12—C1	53.7 (3)	C12—C10—C11—F11B	−130.4 (7)
Al3—Al1—O12—C1	−132.4 (3)	O31—C10—C12—F12B	168.0 (14)
Al2—Al1—O12—C1	176.5 (3)	C11—C10—C12—F12B	27.9 (17)
O11—Al1—O12—Al2	−169.65 (9)	O31—C10—C12—F123	−153.4 (4)
O13—Al1—O12—Al2	84.96 (10)	C11—C10—C12—F123	66.5 (7)
O11^i^—Al1—O12—Al2	−83.00 (10)	O31—C10—C12—F121	−17.9 (7)
O1—Al1—O12—Al2	6.29 (9)	C11—C10—C12—F121	−158.0 (5)
Al1^i^—Al1—O12—Al2	−122.78 (8)	O31—C10—C12—F122	92.0 (4)
Al3—Al1—O12—Al2	51.12 (7)	C11—C10—C12—F122	−48.1 (5)
O32—Al3—O13—Al1	169.07 (12)	O31—C10—C12—F12C	−45.6 (8)
O23—Al3—O13—Al1	56.55 (15)	C11—C10—C12—F12C	174.3 (7)
O31—Al3—O13—Al1	−79.12 (13)	O31—C10—C12—F12A	−122.6 (5)
O1—Al3—O13—Al1	−12.88 (10)	C11—C10—C12—F12A	97.3 (6)
Al4—Al3—O13—Al1	−37.71 (12)	Al3—O32—C13A—C14	−65.1 (7)
Al2—Al3—O13—Al1	22.70 (11)	Al3—O32—C13A—C15	60.9 (7)
O11—Al1—O13—Al3	173.32 (11)	Al3—O32—C13B—C15	110.9 (8)
O12—Al1—O13—Al3	−66.38 (13)	Al3—O32—C13B—C14	−117.3 (6)
O11^i^—Al1—O13—Al3	86.1 (2)	O32—C13A—C14—F143	175.5 (4)
O1—Al1—O13—Al3	13.96 (11)	C15—C13A—C14—F143	50.4 (5)
Al1^i^—Al1—O13—Al3	149.05 (9)	O32—C13A—C14—F141	−59.6 (5)
Al2—Al1—O13—Al3	−22.95 (11)	C15—C13A—C14—F141	175.3 (4)
O23—Al2—O21—C4	124.0 (5)	O32—C13A—C14—F142	55.2 (5)
O51^i^—Al2—O21—C4	−55.2 (5)	C15—C13A—C14—F142	−70.0 (4)
O12—Al2—O21—C4	−144.9 (5)	C15—C13B—C14—F14C	172.2 (13)
O22—Al2—O21—C4	34.1 (5)	O32—C13B—C14—F14C	46.8 (14)
Al1—Al2—O21—C4	−143.4 (4)	C15—C13B—C14—F14B	−44.5 (15)
Al3—Al2—O21—C4	131.7 (5)	O32—C13B—C14—F14B	−169.9 (9)
Al4—Al2—O21—C4	28.8 (6)	C15—C13B—C14—F14A	57.7 (13)
O41—Al4—O22—C7	−0.7 (3)	O32—C13B—C14—F14A	−67.7 (9)
O1—Al4—O22—C7	173.0 (2)	O32—C13B—C15—F15B	58.7 (11)
O52^i^—Al4—O22—C7	−101.4 (2)	C14—C13B—C15—F15B	−62.9 (14)
O31—Al4—O22—C7	96.8 (2)	O32—C13B—C15—F15C	−175.3 (8)
Al3—Al4—O22—C7	129.7 (2)	C14—C13B—C15—F15C	63.2 (14)
Al2—Al4—O22—C7	−178.3 (3)	O32—C13B—C15—F15A	−66.3 (11)
O41—Al4—O22—Al2	177.56 (11)	C14—C13B—C15—F15A	172.1 (10)
O1—Al4—O22—Al2	−8.74 (9)	O32—C13A—C15—F151	169.6 (4)
O52^i^—Al4—O22—Al2	76.86 (11)	C14—C13A—C15—F151	−63.3 (5)
O31—Al4—O22—Al2	−84.97 (11)	O32—C13A—C15—F153	48.2 (4)
Al3—Al4—O22—Al2	−51.99 (8)	C14—C13A—C15—F153	175.4 (4)
O32—Al3—O23—Al2	179.58 (13)	O32—C13A—C15—F152	−68.7 (4)
O13—Al3—O23—Al2	−66.77 (15)	C14—C13A—C15—F152	58.5 (4)
O31—Al3—O23—Al2	68.92 (14)	Al4—O41—C16A—C17	−89.5 (5)
O1—Al3—O23—Al2	2.38 (11)	Al4—O41—C16A—C18	144.6 (3)
Al1—Al3—O23—Al2	−35.82 (11)	Al4—O41—C16B—C17	15 (3)
Al4—Al3—O23—Al2	39.02 (11)	Al4—O41—C16B—C18	144.7 (13)
O21—Al2—O23—Al3	170.11 (13)	O41—C16A—C17—F173	59.7 (6)
O51^i^—Al2—O23—Al3	−13.0 (5)	C18—C16A—C17—F173	−178.6 (5)
O1—Al2—O23—Al3	−2.52 (11)	O41—C16A—C17—F172	−178.0 (4)
O12—Al2—O23—Al3	73.88 (12)	C18—C16A—C17—F172	−56.2 (6)
O22—Al2—O23—Al3	−76.98 (12)	O41—C16A—C17—F171	−58.3 (5)
Al1—Al2—O23—Al3	35.36 (11)	C18—C16A—C17—F171	63.5 (5)
Al4—Al2—O23—Al3	−38.94 (11)	O41—C16B—C17—F17C	169.1 (14)
O32—Al3—O31—C10	18.4 (3)	C18—C16B—C17—F17C	40.3 (19)
O23—Al3—O31—C10	130.1 (3)	O41—C16B—C17—F17B	−40.6 (16)
O13—Al3—O31—C10	−95.5 (3)	C18—C16B—C17—F17B	−169.4 (11)
O1—Al3—O31—C10	−162.4 (3)	O41—C16B—C17—F17A	60.9 (12)
Al1—Al3—O31—C10	−134.0 (3)	C18—C16B—C17—F17A	−67.9 (12)
Al4—Al3—O31—C10	−179.0 (3)	O41—C16A—C18—F181	63.8 (5)
Al2—Al3—O31—C10	164.8 (3)	C17—C16A—C18—F181	−60.4 (6)
O32—Al3—O31—Al4	−162.61 (11)	O41—C16A—C18—F183	−57.6 (6)
O23—Al3—O31—Al4	−50.91 (14)	C17—C16A—C18—F183	178.2 (5)
O13—Al3—O31—Al4	83.45 (12)	O41—C16A—C18—F182	−176.6 (4)
O1—Al3—O31—Al4	16.56 (9)	C17—C16A—C18—F182	59.2 (6)
Al1—Al3—O31—Al4	44.96 (10)	O41—C16B—C18—F18A	−52.7 (14)
Al2—Al3—O31—Al4	−16.17 (10)	C17—C16B—C18—F18A	78.6 (15)
O23—Al3—O32—C13A	34.2 (6)	O41—C16B—C18—F18C	168.5 (11)
O13—Al3—O32—C13A	−84.4 (6)	C17—C16B—C18—F18C	−60.2 (15)
O31—Al3—O32—C13A	154.5 (6)	O41—C16B—C18—F18B	58.6 (12)
Al1—Al3—O32—C13A	−73.3 (6)	C17—C16B—C18—F18B	−170.1 (11)
Al4—Al3—O32—C13A	137.4 (5)	Al5—O53—C19—C21	−35.6 (5)
Al2—Al3—O32—C13A	34.6 (7)	Al5—O53—C19—C20	88.7 (4)
O23—Al3—O32—C13B	37.6 (6)	O53—C19—C20—F202	178.9 (3)
O13—Al3—O32—C13B	−81.0 (6)	C21—C19—C20—F202	−57.3 (4)
O31—Al3—O32—C13B	157.9 (6)	O53—C19—C20—F201	59.0 (4)
Al1—Al3—O32—C13B	−69.9 (6)	C21—C19—C20—F201	−177.2 (3)
Al4—Al3—O32—C13B	140.8 (6)	O53—C19—C20—F203	−59.8 (4)
Al2—Al3—O32—C13B	38.0 (6)	C21—C19—C20—F203	64.0 (4)
O22—Al4—O41—C16B	−151 (2)	O53—C19—C21—F211	−173.3 (4)
O1—Al4—O41—C16B	57 (2)	C20—C19—C21—F211	64.0 (5)
O52^i^—Al4—O41—C16B	−44 (2)	O53—C19—C21—F213	63.5 (4)
O31—Al4—O41—C16B	103 (2)	C20—C19—C21—F213	−59.2 (4)
Al3—Al4—O41—C16B	110 (2)	O53—C19—C21—F212	−51.8 (4)
Al2—Al4—O41—C16B	−147 (2)	C20—C19—C21—F212	−174.5 (3)

Symmetry code: (i) −*x*, −*y*+1, −*z*+1.

### Hydrogen-bond geometry (Å, º)

**Table:**

*D*—H···*A*	*D*—H	H···*A*	*D*···*A*	*D*—H···*A*
O13—H13···O53	0.80 (2)	2.44 (3)	3.080 (3)	138 (4)
O13—H13···F15*A*	0.80 (2)	2.63 (4)	3.094 (13)	119 (3)
O13—H13···F201	0.80 (2)	2.57 (3)	3.266 (3)	146 (4)
O23—H23···F142	0.81 (2)	2.21 (4)	2.876 (4)	139 (5)
O51—H51···F53^i^	0.80 (2)	2.07 (2)	2.850 (3)	163 (5)
O52—H52···F173^i^	0.81 (2)	2.21 (4)	2.841 (6)	136 (5)
O52—H52···F17*A*^i^	0.81 (2)	2.15 (4)	2.806 (12)	139 (5)
O52—H52···F17*B*^i^	0.81 (2)	2.58 (5)	3.123 (19)	126 (4)
C1—H1*A*···O21	1.00	2.48	3.103 (4)	120
C4—H4*A*···F81	1.00	2.32	3.023 (5)	126
C4—H4*A*···F93	1.00	2.52	3.265 (5)	131
C7—H7*A*···O41	1.00	2.59	3.204 (5)	120
C7—H7*A*···F183	1.00	2.43	3.336 (6)	151
C10—H10*A*···O41	1.00	2.19	2.910 (5)	127
C13*A*—H13*A*···F51^ii^	1.00	2.32	3.171 (5)	142
C13*B*—H13*B*···O23	1.00	2.51	3.090 (14)	116
C16*B*—H16*B*···F12*A*	1.00	2.19	2.969 (18)	133

Symmetry codes: (i) −*x*, −*y*+1, −*z*+1; (ii) *x*, *y*+1, *z*.
